# Polio-Free Certification and Lessons Learned — South-East Asia Region, March 2014

**Published:** 2014-10-24

**Authors:** Sunil Bahl, Rakesh Kumar, Nata Menabde, Arun Thapa, Jeffrey McFarland, Virginia Swezy, Rudolph H. Tangermann, Hamid S. Jafari, Linda Elsner, Steven G.F. Wassilak, Olen M. Kew, Stephen L. Cochi

**Affiliations:** 1 National Polio Surveillance Project, World Health Organization; 2 Ministry of Health and Family Welfare, Government of India; 3 World Health Organization Country Office; 4 Immunization and Vaccine Development Department, South-East Asia Regional Office, New Delhi, India; 5 Polio Eradication Department, World Health Organization, Geneva, Switzerland; 6 Global Immunization Division, Center for Global Health, CDC; 7 Division of Viral Diseases, National Center for Immunization and Respiratory Diseases, CDC

In 1988, the World Health Assembly resolved to interrupt wild poliovirus (WPV) transmission worldwide. By 2006, the annual number of WPV cases had decreased by more than 99%, and only four remaining countries had never interrupted WPV transmission: Afghanistan, India, Nigeria, and Pakistan (1). The last confirmed WPV case in India occurred in January 2011 (2), leading the World Health Organization (WHO) South-East Asia Regional Commission for the Certification of Polio Eradication (SEA-RCC) in March 2014 to declare the 11-country South-East Asia Region (SEAR), which includes India,* to be free from circulating indigenous WPV. SEAR became the fourth region among WHO’s six regions to be certified as having interrupted all indigenous WPV circulation; the Region of the Americas was declared polio-free in 1994 (3), the Western Pacific Region in 2000 (4), and the European Region in 2002 (5). Approximately 80% of the world’s population now lives in countries of WHO regions that have been certified polio-free. This report summarizes steps taken to certify polio eradication in SEAR and outlines eradication activities and lessons learned in India, the largest member state in the region and the one for which eradication was the most difficult.

## Steps Toward Regional Certification

Certification of polio eradication is conducted by WHO regions (1,3–5). The Regional Certification Commission (RCC) is an independent body that certifies a region polio-free when all countries in the region meet three conditions: 1) the absence of indigenous WPV transmission for at least 3 consecutive years, monitored by a sensitive, certification-standard surveillance system; 2) the capacity to detect, report, and rapidly respond to any imported WPV; and 3) documentation of substantial progress toward the eventual laboratory containment (at an appropriate biosafety level) of WPV. Each country has an independent National Certification Committee for Polio Eradication to verify and submit country documentation related to polio eradication activities.

**FIGURE 1:**
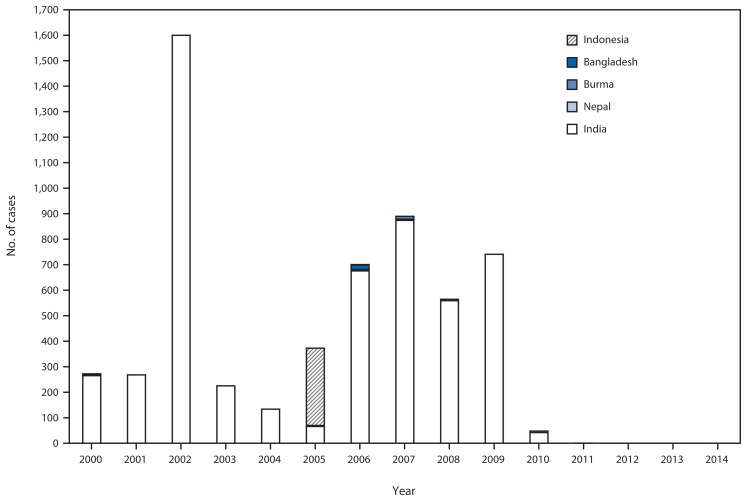
Number of confirmed polio cases from wild poliovirus (WPV) transmission, by country — World Health Organization South-East Asian Region, 2000–2014^*^ ^*^Cases after 2000 in Bangladesh, Burma, and Nepal were associated with WPV imported from India; a 2005–2006 outbreak in Indonesia was associated with WPV type 1 originating in Nigeria.

SEA-RCC, comprised of experts in public health, epidemiology, virology, clinical medicine, and related specialties, reviewed the documentation for each SEAR country. The last confirmed indigenous WPV cases in SEAR countries were as follows: Nepal, 2000; Bangladesh, 2000, Burma, 2000; Thailand, 1997; North Korea, 1996; Timor-Leste, 1995 (recognized as independent in 2002); and Indonesia, 1995. Bhutan, Maldives, and Sri Lanka reported their last cases of polio before 1995. India was the final country in the region to successfully interrupt indigenous WPV transmission, reporting the most recent indigenous WPV type 1 (WPV1) case in SEAR in January 2011. Importation of WPV and subsequent spread occurred in four countries after their last indigenous cases: Nepal reported 26 importation-associated cases during 2005–2010, and outbreaks occurred in Indonesia during 2005–2006 (351 cases), Bangladesh in 2006 (18 cases), and Burma during 2006–2007 (11 cases) (Figure 1). In SEAR, the last identified WPV type 2 (WPV2) case occurred in India in October 1999; this was also the last WPV2 case reported globally. The most recent WPV type 3 (WPV3) case in India and the region occurred in October 2010. After careful review of all documentation, SEA-RCC certified that every SEAR country had met the requirements, and the region was declared polio-free on March 27, 2014 (6).

## Immunization Activities in India

Using the most recent population-based survey data available, India estimated nationwide coverage with 3 doses of oral poliovirus vaccine (OPV) delivered by routine childhood immunization services to be 70.4% among children aged 12–23 months during 2009–2010. Routine coverage estimates in Bihar (61.6%) and Uttar Pradesh (53.9%), two polio-endemic states, were among the lowest in the country (7). Estimated routine coverage improved substantially from estimated levels reported in 2006 in Bihar (47.6%) and Uttar Pradesh (43.8%).

Supplemental immunization activities (SIAs)† in India were introduced as National Immunization Days (NIDs) in 1995, targeting children aged <3 years. NIDs during subsequent years targeted children aged <5 years. NIDs were reinforced by subnational immunization days and large-scale mop-up activities in endemic and other high-risk areas, as well as by introducing house-to-house vaccination as part of the efforts to identify and vaccinate children who were not being brought to the fixed sites providing OPV during SIAs. Since 2000, during NIDs, more than 2.3 million vaccinators visited approximately 209 million households to vaccinate more than 170 million children aged <5 years. Also, surveillance and monitoring data were used to identify high-risk populations and areas for which innovative strategies were designed and implemented. These included strategies to reach mobile and transitory populations by stationing vaccinators at bus stops and train stations, on trains, and at important road intersections, as well as in markets, in migrant camps, at brick kilns, and at construction sites. Transit teams vaccinated nearly 10 million children in each campaign, more than 100,000 of them while on trains. Innovative strategies also were designed to reach children in access-compromised areas in the Kosi river flood plain in Bihar state and in the traditionally highest-risk areas in western Uttar Pradesh and central Bihar. Monitoring data§ were used to estimate the proportion of children missed in each SIA; since 2010, SIAs in India are estimated to have achieved >95% coverage among targeted children, even in remote areas (2).

**FIGURE 2:**
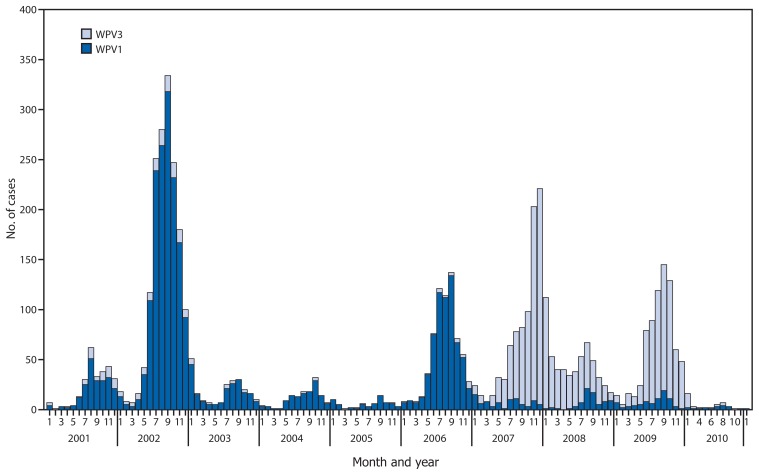
Number of polio cases from wild poliovirus types 1 (WPV1) and 3 (WPV3), by month — India, January 2001–January 2011

India introduced monovalent OPV type 1 (mOPV1) and type 3 (mOPV3) in 2005 (8). Predominant use of mOPV1 greatly reduced the incidence of WPV1 but allowed increased WPV3 incidence until bivalent OPV types 1 and 3 (bOPV) was introduced in 2010 (9). The intensification of the migrant and transit strategies coupled with predominant use of bOPV was associated with a reduction in both WPV1 and WPV3 (Figure 2).

## Wild Poliovirus Surveillance in India

### Acute flaccid paralysis (AFP) surveillance

To demonstrate that WPV circulation has been interrupted, the global standard in endemic countries for sensitive AFP surveillance is an annual rate of at least two nonpolio AFP (NPAFP)¶ cases per 100,000 children aged <15 years. In India, the national NPAFP rate was 13.9 per 100,000 children aged <15 years in 2012 and 12.5 per 100,000 in 2013. The highest state-level NPAFP rates were in Bihar (34.2) and Uttar Pradesh (21.5) Adequate stool specimen collection in India was 87% nationally in 2012 and 86% in 2013, exceeding the performance standard of 80%.

What is already known on this topic?

Extensive regions of the world were certified as free from indigenous wild poliovirus (WPV) transmission during 1994–2002. Until 2011, only four countries remained that had never interrupted WPV transmission: Afghanistan, India, Nigeria, and Pakistan. These four countries are located in three World Health Organization (WHO) regions. WPV exportations from these reservoirs have occurred into many countries that had previously interrupted their indigenous WPV transmission.

What is added by this report?

India, a member of the WHO South-East Asia Region, had its last WPV case in January 2011, which was also the last case in the region. In March 2014, the South-East Asia Region was declared to be free from circulating indigenous WPV and became the fourth WHO region to be so certified. About 80% of the world’s population now lives in countries of regions that have been certified as polio-free.

What are the implications for public health practice?

Stopping indigenous WPV transmission in India required commitment at every level of government, provision of adequate fiscal and human resources, implementation of innovative strategies and approaches, and engagement with the private sector. Specific lessons learned have been successfully applied to address the challenges to polio eradication activities in other countries and are being used to improve immunization services in India.

### Environmental surveillance

Systematic (weekly or biweekly) testing of wastewater samples for poliovirus began in Mumbai, Maharashtra state, in June 2001, in Delhi in May 2010, in Patna, Bihar, in April 2011, in Kolkata, West Bengal, in December 2011, and expanded further to include sampling in the states of Punjab and Gujarat in 2013. Both WPV1 and WPV3 were detected in wastewater sampled at Delhi sites during 2010; WPV3 was most recently detected in July 2010, and WPV1 was most recently detected in August 2010. The most recent WPV isolated from wastewater in India was WPV1 sampled in November 2010 in Mumbai. No WPV has ever been isolated from wastewater sampled in Patna, Kolkata, Punjab, or Gujarat. All WPV1 and WPV3 isolates from wastewater during 2010 were closely related to WPV1 and WPV3 circulating in central Bihar state during 2009.

### Discussion

The certification of SEAR as polio-free conclusively demonstrated that polio can be eradicated in the most challenging settings where the risks for WPV transmission are highest, namely in countries with 1) high population densities, 2) large birth cohorts, 3) high population mobility, 4) poor sanitation, and 5) tropical/subtropical climates. Although such conditions prevail in parts of several SEAR countries, the magnitude and intensity of risks were greatest in India, especially in Uttar Pradesh and Bihar, which have a combined population of approximately 300 million, a monthly birth cohort of approximately 500,000, frequent migration between and outside the states, and lower per-dose effectiveness of trivalent OPV (8,9). Stopping indigenous WPV transmission in India required sustained commitment at every level of government, the provision of adequate fiscal and human resources, the implementation of innovative strategies and approaches, and private sector engagement. Specific lessons learned about ensuring the success of immunization programs throughout India have been successfully applied to address the challenges to polio eradication activities in other countries and are being used to improve immunization services in India (Box).

Strong surveillance is essential for polio eradication. In India, highly sensitive AFP surveillance was supported by a national network of eight fully accredited laboratories capable of basic and advanced molecular virologic detection methods to provide near real-time genetic information, and supplemented by environmental surveillance at key sites. The surveillance system, including the laboratory component, operated highly effectively, as evidenced by performance that consistently surpassed the WHO-recommended standards for global indicators.

Many national and international partners took part in the effort in India. Multiple funding partners helped to supplement the substantial financial investment made to the polio eradication initiative by the Government of India.** Volunteers and community mobilizers played a huge role in the success of India’s eradication efforts, especially to identify the country’s newborns and track their immunization status. In particular, local Rotary International members provided volunteers and funding and engaged with local and national officials to advocate for the country’s immunization programs, including current efforts to improve the routine immunization of infants and young children. The complementary partnership among government and international polio implementing partners contributed to the success of the India polio eradication program.

BOX **Critical lessons learned from India’s polio eradication effort**

- **Engage every level of government and make local authorities accountable.** District administrators (“magistrates”) led task forces to review supplemental immunization activity (SIA) planning and implementation and ensured that all district government sectors were involved in the program.
- **Develop robust communication strategies to ensure program effectiveness.**
- **Optimize vaccination team composition and ensure objective supervision**. Vaccination teams should include at least one female and one member from the local community to facilitate entry into households.
- **Develop and validate detailed plans (“microplans”).** Microplans in India, in which all houses in the area were numbered and realistic workloads established for each vaccination team, were regularly validated and updated. Meticulous planning and implementation of SIAs led to high coverage, even in areas with weak health systems.
- **Accurately monitor data on campaign quality in real time and assess coverage independently at the end of each round.** Monitoring data can drive immediate corrective actions and ensure accountability.
- **Engage the private sector to increase program visibility and reach maximal impact.**
- **Innovate to identify and vaccinate children who were previously being missed.** Significant innovations, strategies, and tactics used in India’s immunization program included the following:
  – Engaging community and religious leaders in planning and implementing SIAs in areas with reluctant participants.
  – Instituting finger marking of vaccinated children to help identify those not yet vaccinated and marking the dwellings of households visited by vaccination teams to increase the likelihood of follow-up.
  – Identifying and tracking newborns.
  – Targeting high-risk areas with multiple health interventions and additional resources.
  – Implementing a strategy for reaching children at public gatherings and in mobile and transitory populations (10).
- **Conduct research to help overcome technical and operational barriers.** Technical research led to introduction of more efficacious vaccines (i.e., monovalent OPV in 2005 and bivalent OPV in 2010). Research included seroprevalence and immunogenicity studies and operational studies such as social network analysis to provide evidence for decision-making.

## Footnotes

* Bangladesh, Bhutan, Burma, Indonesia, India, Maldives, Nepal, North Korea, Sri Lanka, Thailand, and Timor-Leste.

† Mass campaigns conducted during a short period (days to weeks), during which a dose of OPV is administered to all children (generally those aged <5 years) regardless of previous vaccination history. Campaigns can be conducted nationally or in portions of the country (i.e., subnational SIAs).

§ SIA monitoring data are obtained from systematic surveys conducted after every SIA in high-risk areas to identify children aged <5 years who were missed with vaccination.

¶ GPEI sets operational targets for countries with current or recent WPV transmission, both nationally and in each province or state. Targets are NPAFP ≥2 per 100,000 population aged <15 years per year, and adequate stool specimen collection from ≥80% of AFP cases, in which two specimens are collected ≥24 hours apart, both within 14 days of paralysis onset, shipped on ice or frozen ice packs, and arriving in good condition (without leakage or desiccation) at a WHO-accredited laboratory.

** Major international partners include WHO, Rotary International, the World Bank, the Bill and Melinda Gates Foundation, UNICEF, and the governments of Germany, Japan, the United Kingdom, and the United States, including CDC.
